# New Delhi Metallo – beta lactamase – 1 containing Enterobacteriaceae: Origin, Diagnosis, Treatment and Public health concern

**Published:** 2012-02-13

**Authors:** Jaykaran Charan, Summaiya Mulla, Sridhar Ryavanki, Naresh Kantharia

**Affiliations:** 1 Department of Pharmacology, Govt. Medical College, Surat, India; 2 Department of Microbiology, Govt. Medical College, Surat, India; 3 Department of Community Medicine, Govt. Medical College, Surat, India

**Keywords:** Antibiotics resistance, Enterobacteriaceae, New Delhi Beta metallo lactamase, carbapenemases

## Abstract

One of the biggest problems associated with the antibiotic therapy is resistance. Recently published studies have revealed that enterobacteriaceae, like E. coli and Klebsiella, isolated from several Indian centers are resistant to many antibiotics including some highly potent antibiotics like carbapenems. It has been proposed that this resistance is because of a carbapenemase enzyme called NDM- 1 (New Delhi Metallo-betalactamase-1). This carbapenemase is class B carbapenemase also called metallolactamases as they require zinc at their active site. This enzyme is coded by a gene called bla - NDM -1 or gene NDM -1. NDM -1 containing enterobacteriaceae can be screened in laboratory by few techniques. Metallolactamase production can be detected by disk approximation test or Modified Hodge test and NDM -1 gene can be detected by polymerase chain reaction by the use of specific primer targeting the gene. Infections caused by such bacteria are associated with high morbidity and mortality. Two classes of antibiotics i.e., polymyxins (colistin) and glycylcyclines (tigecyclines), have shown in vitro activity against NDM -1 harboring enterobacteriaceae. The safety profile of both of these antibiotics is questionable. There is a need for active screening of microorganisms for NDM -1 and research should be directed towards the development of safe antibiotics for the treatment of these kinds of infections.

## Introduction

Microorganisms existed on this earth millions of years before the evolution of man. Revolution in the medical sciences during the 19th century, along with the advent of antibiotics aroused a hope to do away with the microorganisms of concern to man, but today microbial resistance to antibiotics continues to be a serious threat to mankind. A number of articles published in indexed journals predict that, in near future, we may encounter a group of entirely new bacteria which may not be amenable to any of the presently available antibiotics This could have a tremendous impact on the advanced medical procedures like organ transplantation, bone marrow transplantation and cancer chemotherapy [1]. Kumarasamy et al, in a study published in Lancet Infectious Disease journal, reported that enterobacteriaceae isolated from two centers in India were highly resistant to all antibiotics except colistin and tigecycline.

Majority of these organisms carried a novel gene on their plasmid which was named as New Delhi Metallo-betalactamase (NDM -1, bla NDM-1). Kumarasamy et al also reported that isolates from Haryana had a similar genetic finger print. This is indirect evidence for their common origin and, hence a potential epidemic threat [2]. Walsh T et al, in a study published in Lancet Infectious Disease journal, found that seepage and tap water collected from New Delhi contained microorganisms resistant to all known antibiotics except colistin and tigecycline which contain bla NDM -1 gene [3].

These studies were received with criticism. There were issues related to the naming of the gene after an Indian city, invalid methodology, and illogical conclusion [4–8]. In the midst of criticism and controversy, one cannot deny the fact regarding existence of a “superbug”, resistant to all known antibiotics and the need to prevent the rapid spread of such organisms. Though the information existed previously, the awareness of NDM-1 containing microorganisms only came about after the publication of Kumarasamy et al's study [2]. A PubMed and Google search was done using MeSH term “NDM -1” or “New Delhi Metallolactamase-1”. The authors found 12 articles (Table 1) reporting NDM-1 gene containing bacteria (reviews, letters, articles related to laboratory investigations were excluded) [9–18].

**Table 1:** Studies reporting NDM- 1 containing enterobacteriaceae

No.	Authors	Outcome
1	Yong et al [9] (2009)	Case report of a Swedish patient of Indian origin, who acquired urinary tract infection by carbapenem resistant *Klebsiella pneumoniae* during visit to New Delhi (India). The organism was found to contain NDM -1 gene on plasmid.
2	Deshpande et al [10] (2010)	Out of 24 carbapenem resistant enterobacteriaceae collected over three months in a tertiary care centre in Mumbai, 22 were found to have NDM -1 gene.
3	Karthikeyan et al [11] (2010)	Case report of three isolates of carbapenem resistant *A. baumannii*, isolated from tertiary care centre at Chennai, reporting coexistence of three genes: bla OXA -23, bla NDM -1 and armA, responsible for resistance to various antibiotics.
4	Kumarasamy et al [2] (2010)	NDM -1 gene containing enterobacteriaceae from 44 samples in Chennai, 26 in Haryana, 37 in United Kingdom and 77 in other Indian cities.
5	Poirel et al [12] (2010)	Case report of a 67 year old man, who was hospitalized in Sydney (Australia), and had been to Bangladesh just before coming to Australia for treatment. In Bangladesh, he was treated for pneumonia. *E. Coli* isolated from his urine was multi drug resistant and contained NDM -1 gene on plasmid.
6	Wu et al [13] (2010)	Case report of a 38 year old Taiwanese man, who had suffered a gunshot injury in New Delhi and got treatment for the same in New Delhi for 8 days. He came back to Taipei for further treatment. NDM -1 carrying multidrug resistant *Klebsiella pneumoniae* was found in two consecutive fecal sample of that patient.
7	Struelens et al [14] (2010)	77 cases of NDM -1 positive enterobacteriaceae was reported in 13 European countries in 2008 – 2010.
8	Chihara et al [15] (2011)	Case report of a Japanese man, who had travelled to India for business. In India, he developed Guillain-Barre syndrome and was treated for the same in local hospital. He returned Japan and was admitted in a hospital for further treatment. NDM – 1 carrying E. Coli was found in his blood sample.
9	Mulvey et al [16] (2011)	Multidrug resistant *Klebsiella pneumoniae* and Escherichia coli containing NDM – 1 gene were isolated from a patient who had returned back to Canada from India.
10	Zarfel et al [17] (2011)	Case report of isolation of NDM -1 positive *Klebsiella pneumoniae* in a patient in Austria, who had a treatment history in India and Pakistan.
11	Sidjabat et al [18] (2011)	Case report of an 87 year old Australian resident of Indian origin, who was admitted in an Australian hospital for chronic draining foot ulcer for which he had taken treatment from India. *Klebsiella pneumoniae* isolated from her urine was resistant to carbapenem and found to be harboring NDM -1 gene.
12	Walsh et al [3] (2011)	NDM -1 positive enterobacteriaceae found in seepage and tap water collected from area around central New Delhi. Similar samples taken from Cardiff found no such gene on the microorganisms.

A recently published surveillance study discovered, that enterobacteriaceae containing NDM-1 were present in New Delhi from as early as 2006 [19]. But, the first significant study addressing the problem of NDM-1 gene containing multidrug resistant microorganism was a case report by Yong et al [9]. In this case report, carbapenem resistant Klebsiella pneumoniae, isolated from the urine of a 59 year old Swedish resident of Indian origin, was found to be harboring NDM-1 gene. The patient was suffering from type 2 diabetes with a history of multiple strokes. During his stay in India, he was operated for gluteal abscess at Ludhiana (Punjab), and for decubital ulcer at New Delhi.

The problem was again highlighted by Deshpande et al in a study published in Journal of Association of Physicians of India [10]. In this study, out of 24 carbapenem resistant Enterobacteriaceae recovered from a tertiary hospital, 22 were found to be harboring NDM -1 gene. Out of these 22 NDM-1 positive Enterobacteriaceae, 10 were Klebsiella spp, 9 Escherichia coli, 2 Enterobacter spp and one isolate of Monganella morganii. NDM -1 positive Enterobacteriaceae were also reported from other countries among patients, who had travelled to India, Pakistan and Bangladesh [11–18]. This caused controversy after the publication of Kumarasamy et al [2] and Walsh et al [3], reporting NDM-1 harboring Enterobacteriaceae in Indian patients and water in New Delhi respectively.

### What is NDM-1?

NDM-1(New Delhi Metallo-betalactamase-1) is a carbapenemase beta lactamase enzyme. This enzyme is coded by blaNDM -1or NDM-1 gene. This gene encodes, 269 amino acids containing protein, with molecular mass of approx 27.5 kDa [1]. Carbapenemases are beta lactamase which can hydrolyze the beta lactams, particularly carbapenem. They are resistant to clavulanic acid, salbactum and many other commercially available beta lactamase inhibitors.

Broadly carbapenemases are categorized into four groups; Class A: Penicillinases, Class B: Metallolactamases, Class C: Cephalosporinase or ampC and Class D: Oxacillinases [20]. NDM -1 belongs to class B- Carbapenemase. The characteristic property of class B carbapenemase is that, they require zinc at their active site; hence they are called as metallolactamases. The activity of these enzymes can be inhibited by Zinc chelating agents like EDTA [21]. Metallolactamases also hydrolyze cephalosporins and penicillins. Aztreonam resist hydrolysis by metallolactamases [21]. The bla NDM -1 gene is accompanied by other genes responsible for resistance to antibiotics, like erythromycin, ciprofloxacillin, rifampicin and chloramphenicol. It is also accompanied by a broad spectrum beta lactamase (CMY -4), also a genetic element, which encodes for efflux pump and its promoters. All these accompanying genes make bla NDM -1 more dangerous in causing multidrug resistance [22].

In the Kumarasamy et al study, it was noted that blaNDM -1 also coexisted with amino glycoside resistant genes like blaOXA- 23 and armA [11]. A similar study by Poirel et al, observed that the NDM-1 gene in a strain of Citrobacter freundii was accompanied by 9 different types of beta lactamase [23]. The blaNDM -1 gene is located on plasmid, which can be easily transferred to other bacteria by conjugation, and it is found to be located on plasmid of different sizes, which predicts its mobility and pathogenesis [24]. NDM -1 positive organism can get colonized in the gut in the absence of any feature of disease. As most of these organisms are normal commensals of the gut, the screening of these organisms is a difficult task [24].

## Laboratory detection of NDM -1

Laboratory detection of NDM -1 involves: 1) Identification of the microorganism, 2) Detection of metello -beta- lactamase production and 3) Identification of bla NDM -1 [2,9,10].

Identification of bacteria can be done by automated systems like Phoenix or by API 20 E Strips [2]. Elevated Minimum Inhibitory Concentration (MIC) to carbapenem is the foremost event that can indicate the carbapenemase (metallo-beta lactamase and other) production [21]. Elevated MIC predicts carbapenemase production in enterobacteriaceae but it cannot predict total clinical resistance [21]. MIC can be measured by microbroth dilution, agar dilution or by disk diffusion. According to recent guideline by Clinical Laboratory Standards Institute (CLSI), if an organism is resistant to carbapenem in accordance with CLSI cut off points, then there is no further need to go for phenotype testing by other methods like modified hodge. This holds well not only for clinical reporting, but for epidemiological and research purposes. Carbapenem resistant bacteria detection by MIC method, should be further explored for carbapenemase production by phenotypical methods [25]. It is very important to understand that the variability in susceptibility of cabapenem seems to depend on the type of automated system used [26].

Production of metallo -beta- lactamase can be identified by disk approximation test (or double disk synergy test) using EDTA as metal chelater. In disk approximation test, clear zone (zone of inhibition) around the beta lactum disk, is increased in the presence of zinc chelator like EDTA. It is observed that imipenem-EDTA combination was most sensitive for metallo beta lactamse production [27]. Metallo beta lactamse can also be confirmed by Etest strip. E test strip of imipenem and imipenem-EDTA combination is used to detect metallo-beta-lactamase production. Three fold or greater decrease in MIC of imipenem in the presence of EDTA indicates the production of metallo-beta-lactamase, but there are possibilities of false positivity and false negativity. False negative results, when MIC of imipenem is less than 4 microgram/ml [27]. False positivity results, in the presence of OXA genes or direct action of EDTA on microorganism by changing former's permeability [28]. Modified Hodge test can also be used for the same purpose. This is a phenotypic test for confirming the presence of carbapenemase (metallo-beta lactamase) production. Cloverleaf indentation at the intersection of the test organism and standard strain, within the zone of inhibition of carbapenem disk shows positive result for carbapenemase production [29]. The advantage of this method is that even the weak carbapenemase activity enzyme can be detected [21]. Other methods like isoelectric focusing and imipenem hydrolysis can also be used for the same purpose [21]. Presence of bla NDM-1 can be detected by polymerase chain reaction by the use of specific primer targeting the gene [2,9,10,21].

## Treatment of infection caused by NDM-1 harboring Enterobacteriaceae

Infections caused by such bacteria are associated with high morbidity and mortality. Even though specific data on NDM-1 positive organisms is not available, there is indirect evidence to suggest that carbapenemase producing Enterobacteriaceae infection is associated with high crude and attributable mortality. In a study by Bores A et al, it was observed that crude mortality and attributable mortality in the patients of carbapenemase producing Kliebsella pneumoniae bacteremia was 71.9% and 50% respectively [30]. In a similar study by Patel G et al, it was noted that mortality among the cases with carbapenemase resistant Klebsiella pneumoniae was significantly more as compared to control (40% Vs 20%). Timely administration of in vitro sensitive antibiotics was not associated with survival [31]. Two classes of antibiotics i.e., polymyxins (colistin) and glycylcyclines (tigecyclines), have shown in vitro activity against NDM-1 harboring Enterobacteriaceae. On the basis of in vitro sensitivity analysis of 37 British isolates, 50% and 90% MIC for colistin was 0.5 mg/ L and 8 mg/L respectively ,and for tigecycline, it was 1 mg/L and 4 mg/L [32]. In the Kumarasamy study, it was observed that 89% of UK isolates were susceptible to colistin and 64% to tigecycline. Out of all Indian isolates, more than 50% were sensitive to tigecycline and all were sensitive to colistin [2], but the chances of resistance are also high. In the Kumarasamy et al study one isolate (from Chennai) was highly resistant to all known antibiotics. A similar finding was also observed in studies done in Greece and New York [33,34,35]. Resistance to colistin and tigecycline should be a reason for worry, as it indicates a return to the pre antibiotic era [32]. Along with resistance, one more limitation of drug therapy in such infections is toxicity. Colistin group was practically abandoned 30 years ago due to its nephrotoxicity. In a study by Souli et al, it was observed that, attributable mortality by colistin containing regimen in patient infected by carbapenemase producing Klebsiella pneumoniae was around 19% [33].

Tigecycline is a relatively new antibiotic. In a small number of studies, it was observed that Tigecycline has favorable outcomes in 70% of the patients infected with carbapenem and multidrug resistant organisms. But, a recent US Food and Drug administration update warned about the increase in mortality among patients taking tigecycline as compared to other antibiotics [36]. A combination of tigecycline and colistin has been explored in some studies done for carbapenemase producing Enterobacteriaceae.

Cobo J et al , observed that colistin and tigecycline combination is synergistic [37]. Pournaras et al, on the basis of time kill assay, studied the effect of tigecycline alone, and its combination with meropenam and colistin, on carbapenemase producing Klebsiella pneumoniae strain. It was observed that as a single agent, none of the three antibiotics (tigecycline, meropenam and colistin) showed bactericidal concentration. Tigecycline and colistin, when given together produced bactericidal effect [38]. Some studies also reported the successful treatment of pan resistant Enterobacteriaceae with combination therapy of colistin and tigecycline [39]. Combinations using aztreonam or any other monobactum which are resistant to hydrolysis by metallolactamases are recommended by some studies. Here it is important to understand that NDM-1 containing bacterial strain may also have other carbapenemase like ampC or ESBLs which may hydrolyze the aztreonam. So inhibitors of these enzyme (like NXL104) should be the part of such combination therapy [40]. Fosfomycine is also suggested for use in carbapenemase producing pan resistant Enterobacteriaceae [41].

## Public health concerns and way forward

India has quite a lot of skilled physicians and nurse practitioners. Over the last two decades, the economic boom in India has led to the expansion of infrastructure with medical facilities, at par with the medical care that the west has to offer. While some small countries may be viable as alternatives for minor surgical procedures, India is the only mainstream option that offers a comprehensive solution for any and all medical needs, and it does this with the highest levels of service, facilities, and professional skills. A complex transplant or bypass procedure can be achieved with a fraction of the cost as compared to U.S.A [42].

Several published articles particularly case reports show link, between NDM-1 containing pan resistant Enterobacteriaceae and India. It has been argued, that these “superbugs” originated in Indian subcontinent [2,3,9]. Various factors like over the counter use of antibiotics, irrational use of antibiotics, easy accessibility of higher antibiotics, poor sanitation, high prevalence of diarrhea and overcrowding are considered responsible for development and spread of these organisms [9]. A Lancet Infectious Disease study, warned patients against going to India for medical treatment as they may get infected with these organisms, which ultimately cost more than “the short term saving” [2]. This conclusion was questioned by press, politicians, scientists as well as the first author of the same study [4]. Recent study which shows the presence of NDM-1 harboring organism in New Delhi water, but absence of such organism in Cardiff water, indicates that the problem is more severe in countries like India. Instead of denial, there is a need to understand the severity of the situation, the burden it can pose in future, and to develop the necessary strategies to halt the development and spread of NDM -1. Steps are already being taken in western countries [24,43,44] and similar or more aggressive steps are needed in India.

An active surveillance system encompassing various microorganisms should be put in place. There is a need for an appropriate system by which recent trend of bacterial susceptibility to various antibiotics can be notified at various levels. There is also a need of National Reference Laboratory and Research Institute for systematic study and vigilance of antibiotic resistance. Monitoring of prescription is required to be done regularly in hospitals and irrational or inappropriate use of antibiotics be discouraged. Unethical promotional policies of pharmaceutical companies should be taken seriously as it is duly observed, that many of the claims made in promotional material distributed by pharmaceutical companies, are not supported by valid evidence [45]. Clinicians instead of relying on pharmaceutical companies for information related to any drug including antibiotics, try to find out for themselves through journals or by participating in CMEs, conferences. Knowledge and effective management of locally endemic infectious diseases needs to be given more emphasis in both undergraduate and postgraduate curriculum [1]. Clear cut antibiotic policies in every hospital should be implemented and the use of reserved antibiotics left for cases of utmost need only.

The same has been advocated by the WHO in its 2011 World Health Day theme “Combat Antimicrobial Resistance: No Action Today, No Cure Tomorrow”. Stringent laws to curb selling of over the counter (OTC) antibiotics have to be discussed and implemented at the national level. Facilities for detection of NDM-1 are to be made accessible to all hospitals and some molecular or diagnostic probes should be developed for rapid detection of this gene in bacteria [46]. This is an opportune time for health policy specialists, scientists, academicians and government official to collectively analyse the present situation and take some concrete steps for prevention as well as spread of this resistance.

## Conclusion

Because of the irrational use of antibiotics more and more microorganisms are developing resistance to available antibiotics. Enterobactareaceae class of microorganisms is clinically very important and emergence of resistance to almost all antibiotics in this class should be matter of concern to clinicians and health authorities. Clinicians should be trained about the rational use of antibiotics and legislative changes should be done to prevent widespread and irrational use of antibiotics.
